# Correction to: Applying a Precautionary Approach to Mobile Contact Tracing for COVID-19: The Value of Reversibility

**DOI:** 10.1007/s11673-021-10106-2

**Published:** 2021-05-12

**Authors:** Niels Nijsingh, Anne van Bergen, Verina Wild

**Affiliations:** Institute for Ethics, History and Theory of Medicine, Ludwig Maximilians Universität, Lessingstrasse 2, 80336 München, Germany

This paper was previously published without the following funding details: This article was supported by the Bundesministerium für Bildung und Forschung (BMBF), grant number FKZ 01GP1791. The original article has been corrected.

